# The Prevalence of Sickle Cell Disease in Colorado and Methodologies of the Colorado Sickle Cell Data Collection Program: Public Health Surveillance Study

**DOI:** 10.2196/64995

**Published:** 2024-12-09

**Authors:** Joshua I Miller, Kathryn L Hassell, Yvonne Kellar-Guenther, Stacey Quesada, Rhonda West, Marci Sontag

**Affiliations:** 1 Center for Public Health Innovation, 891 Evergreen Parkway, Evergreen, CO, 80439, United States, 1 7204121174; 2 Division of Hematology Colorado Sickle Cell Treatment and Research Center, School of Medicine, University of Colorado Denver, Aurora, CO, United States

**Keywords:** sickle cell disease, public health surveillance, prevalence, birth prevalence, Colorado, sickle cell, surveillance, SCD, USA, data collection, blood disorder, policy development, hematology, United States

## Abstract

**Background:**

Sickle cell disease (SCD) is a genetic blood disorder that affects approximately 100,000 individuals in the United States, with the highest prevalence among Black or African American populations. While advances in care have improved survival, comprehensive state-level data on the prevalence of SCD remain limited, which hampers efforts to optimize health care services. To address this gap, the Colorado Sickle Cell Data Collection (CO-SCDC) program was established in 2021 as part of the Centers for Disease Control and Prevention’s initiative to enhance surveillance and public health efforts for SCD.

**Objective:**

The objectives of this study were to describe the establishment of the CO-SCDC program and to provide updated estimates of the prevalence and birth prevalence of SCD in Colorado, including geographic dispersion. Additional objectives include evaluating the accuracy of case identification methods and leveraging surveillance activities to inform public health initiatives.

**Methods:**

Data were collected from Health Data Compass (a multi-institutional data warehouse) containing electronic health records from the University of Colorado Health and Children’s Hospital Colorado for the years 2012‐2020. Colorado newborn screening program data were included for confirmed SCD diagnoses from 2001 to 2020. Records were linked using the Colorado University Record Linkage tool and deidentified for analysis. Case definitions, adapted from the Centers for Disease Control and Prevention’s Registry and Surveillance System for Hemoglobinopathies project, classified cases as possible, probable, or definite SCD. Clinical validation by hematologists was performed to ensure accuracy, and prevalence rates were calculated using 2020 US Census population estimates.

**Results:**

In 2019, 435 individuals were identified as living with SCD in Colorado, an increase of 16%‐40% over previous estimates, with the majority (n=349, 80.2%) identifying as Black or African American. The median age of individuals was 19 years. The prevalence of SCD was highest in urban counties, with concentrations in Arapahoe, Denver, and El Paso counties. Birth prevalence of SCD increased from 11.9 per 100,000 live births between 2010 and 2014 to 20.1 per 100,000 live births between 2015 and 2019 with 58.5% (n=38) of cases being hemoglobin (Hb) SS or HbSβ^0^ thalassemia subtypes. The study highlighted a 67% (n=26) increase in SCD births over the decade, correlating with the growth of the Black or African American population in the state.

**Conclusions:**

The CO-SCDC program successfully established the capacity to perform SCD surveillance and, in doing so, identified baseline prevalence estimates for SCD in Colorado. The findings highlight geographic dispersion across Colorado counties, highlighting the need for equitable access to specialty care, particularly for rural populations. The combination of automated data linkage and clinical validation improved case identification accuracy. Future efforts will expand surveillance to include claims data to better capture health care use and address potential underreporting. These results will guide public health interventions aimed at improving care for individuals with SCD in Colorado.

## Introduction

Sickle cell disease (SCD) is a hereditary blood disorder characterized by the presence of abnormal red blood cells that assume a rigid, sickle-shaped form, leading to impaired blood flow and various health complications [1]. Recent advancements in health care have significantly improved the survival rates of individuals with SCD, with over 95% of patients born in developed countries expected to survive into adulthood due to enhanced supportive and preventive care measures such as newborn screening (NBS), penicillin prophylaxis, and transcranial Doppler screening [2]. Despite improvements, the disease still poses significant challenges, with potential worsening of complications in adolescence and young adulthood, potentially compounded by reduced adherence to treatment protocols and the need to transition from pediatric to adult care [3]. Mortality rates among adults with SCD have increased over time, possibly indicating limited access to or underuse of high-quality care for this demographic [4].

Estimates from 2010 suggest that the number of individuals affected by SCD in the United States could be 100,000, though limitations in data quality and variations among states hinder precise estimations [5]. In 2010, Hassell [6] published SCD population estimates for the United States derived from the 2008 United States Census and the birth-cohort disease prevalence from the National Newborn Screening Information System (NNSIS). Hassell estimated a NNSIS-based total birth cohort of between 311 and 371 individuals with SCD living in Colorado (CO) between 2005 and 2007, after correcting for early mortality. These prevalence estimates for CO have not been updated since.

The Centers for Disease Control and Prevention initiated the Registry and Surveillance System for Hemoglobinopathies (RuSH) project [7,8] in 2010 to enhance estimates of SCD. This marked the inception of the first comprehensive surveillance initiative for SCD in the United States, incorporating data from multiple sources. Subsequently, the Public Health Research, Epidemiology, and Surveillance for Hemoglobinopathies project [7,8], conducted from 2012 to 2014, aimed to expand the knowledge derived from RuSH data, refining surveillance methodologies, and instituting strategies for health promotion and the prevention of associated health complications.

The Sickle Cell Data Collection (SCDC) program subsequently began in 2015 to expand SCD surveillance activities by collecting diagnosis, treatment, and health care use data on individuals with SCD. Colorado was selected for funding in 2021 and established the Colorado Sickle Cell Data Collection program (CO-SCDC) with the aim of instituting a SCD surveillance system to serve the CO SCD community by providing relevant data that would inform public health interventions, education initiatives, and policy development.

The Center for Public Health Innovation (CPHI) established a multidisciplinary team (MDT) across multiple institutions in 2021 to manage the CO-SCDC program. The MDT is comprised of epidemiologists, health data analysts, program evaluators, and health communication experts at CPHI, and adult and pediatric hematologists at the Colorado Sickle Cell Treatment and Research Center (CSCTRC) at the University of Colorado School of Medicine who are involved with the clinical services provided to individuals with SCD at the Children’s Hospital Colorado (CHCO) and the University of Colorado Health (UCHealth) systems. The MDT also consists of leaders from the Colorado Sickle Cell Association (local community-based organization), representatives from NBS and vital statistics programs in the Colorado Department of Public Health and Environment, a chronic disease surveillance specialist from the Colorado School of Public Health, a mental health professional specializing in pain management at Road to Me Recovery, an employment services expert at the Colorado Department of Vocational Rehabilitation, and a SCD transition specialist social worker from the CSCTRC.

In this paper, we describe the CO-SCDC project, the collaborative partnerships developed in the state, and the methods used to obtain SCD surveillance data within CO. We report updated data on the population and birth prevalence of SCD in individuals living in Colorado in 2019, derived from multiple data sources, the application of case definitions, and subsequent case validation.

## Methods

### Data Acquisition

CO-SCDC acquired linked and deidentified data from Health Data Compass (Compass) [9], the honest broker for the project. Compass is a multi-institutional data warehouse funded by UCHealth, CHCO, and the University of Colorado School of Medicine, and is designed to support data discovery and data science methodologies that integrate, harmonize, and link large-scale biological, clinical, administrative, regional, state, and national data sets. Compass incorporates electronic health record (EHR) data from all health records within the UCHealth and CHCO systems across CO. This includes records from individuals seen across 4 CHCO hospitals and 6 outpatient clinics, and 12 UCHealth hospitals and over 120 outpatient clinics in CO. Compass also receives notification of deaths in the state of Colorado for all individuals in the Compass system through a partnership with the CO Vital Statistics Program.

Health care encounters across all CHCO and UCHealth hospitals and outpatient clinics for individuals with SCD-related *International Classification of Diseases, Ninth Revision, Clinical Modification* (*ICD-9-CM*) [10] and *International Classification of Diseases, Tenth Revision, Clinical Modification* (*ICD-10-CM*) [11] codes from January 1, 2012, through December 31, 2020, were pulled by Compass and deidentified.

The CO Newborn Screening Program [12,13] provided data from January 1, 2001, to December 31, 2020, on infants with a confirmed SCD diagnosis. These data included date of birth, biological sex, mother’s basic demographics, and final diagnostic SCD subtype. To obtain race, ethnicity, and county of residence at birth, NBS data were merged with birth records through the CO Vital Statistics Program. The Vital Statistics Program linked the NBS records with the specific birth record fields of interest. Once linked, the NBS data and birth record data were entered into a REDCap database housed at UCHealth [14,15]. The individual-level, fully identifiable data were pulled by Compass and linked with patient data for individuals with UCHealth and CHCO health care encounters and SCD-related *ICD-9-CM* and *ICD-10-CM* codes.

All data linkages at Compass were performed by using the Colorado University Record Linkage tool to match and merge records for all data listed above. Colorado University Record Linkage is a platform that performs record linkage operations and uses hashing to encode sensitive data into random strings of information ensuring that patient data are secure across all parties involved once the data are linked [16]. All linked identifiable data were stored on secure servers at UCHealth Compass. Data were then deidentified and transferred as limited data sets directly to the CPHI Microsoft Office E5 level Health Information Portability and Accountability Act–compliant OneDrive [17].

Analysts at CPHI cleaned and formatted the data and performed additional deduplication. Demographic fields, dates, diagnostic data, and acute care use fields were reformatted to match across all institutions. Potential data errors were identified and corrected or removed. Arbitrary person identifiers assigned by Compass were compared within and across institutions and deduplicated. Any health care encounters with a county of residence outside of CO were excluded. All data processes were performed using SAS (version 9.4; SAS Institute Inc).

### Case Definitions

After data cleaning, case definitions were applied. Using the case definition from the RuSH project as a basis, we adapted the definitions to determine which individuals represented a case of possible, probable, or definite SCD [7,18,19]. Proof of residence in CO was established for any individual with 3 or more health care encounters across participating hospitals, emergency departments, or outpatient clinics with an SCD *ICD-9-CM* or *ICD-10-CM* code over any 5-year period between January 1, 2012 and December 31, 2019, and were labeled as possible cases. Using acute inpatient care and ED use as indicators of disease, possible cases were upgraded to probable if the individual had a hospital discharge in 2019, an emergency department visit in 2019, or both before and after 2019. A case was confirmed as definite if they had a confirmed diagnosis of SCD after a presumptive positive newborn screen or were confirmed to have SCD via chart review by a clinical hematologist at the CSCTRC. Only probable and definite cases were reported.

### Case Validation

All possible, probable, and definite cases from the year 2018 were submitted to 1 adult and 1 pediatric hematologist at the CSCTRC for case validation. The hematologists went through the EHR and determined whether an individual was a definite case, not a case, or unsure if not enough information was available. All individuals determined to be a definite case were included in the counts for 2019. All individuals determined not to have SCD were removed from the cohort and excluded from the analysis, as were individuals determined to have sickle cell trait. Individuals with a bone marrow transplant (BMT) prior to 2019 were considered cured and excluded.

### Positive and Negative Predictive Value

Positive predictive value (PPV) and negative predictive value (NPV) were calculated to measure the accuracy of the applied case definitions. PPV was calculated by dividing the true positives (TP), or those confirmed to have SCD via case validation, by the total number of cases found using the case definitions. NPV was calculated by dividing the true negatives, or those confirmed not to have SCD via case validation, by the total number of negative cases found using the case definitions.


PPV=TruePositives/(TruePositives+FalsePositives)

NPV=TrueNegatives/(TrueNegatives+FalseNegatives)

### Geographic Prevalence

Frequencies and descriptive statistics were computed for both probable and definite cases. County was assigned based on the county of residence recorded for the individual at the latest health care encounter during the surveillance period. Prevalence rates at the state and county levels were determined by dividing the combined number of definite and probable cases by the estimated population of individuals residing in CO or specific CO counties, as per the 2020 US Census population estimates [20]. These rates were then multiplied by 100,000 to obtain the prevalence per 100,000 individuals. Prevalence numbers by race were calculated similarly by using the total Black or African American population as the denominator.

### Birth Prevalence

The linked NBS and vital record data were used to calculate birth prevalence. Birth prevalence rates were calculated by dividing the number of confirmed SCD births in CO by the total number of live births in CO [21] and then multiplied by 100,000 to obtain birth prevalence rates per 100,000 people. Birth prevalence by race was calculated similarly by using the total Black or African American live births as the denominator. All data analyses were performed using SAS (version 9.4; SAS Institute Inc).

### Ethical Considerations

The CO-SCDC program underwent ethical review by the Colorado Multiple Institutional Review Board and was classified as initial secondary research and granted a waiver of consent (Colorado Multiple Institutional Review Board number 21‐3244). All data provided to us by Compass and the Colorado Department of Public Health and Environment were deidentified.

## Results

### 2018 SCD Case Validation

**Figure 1.**
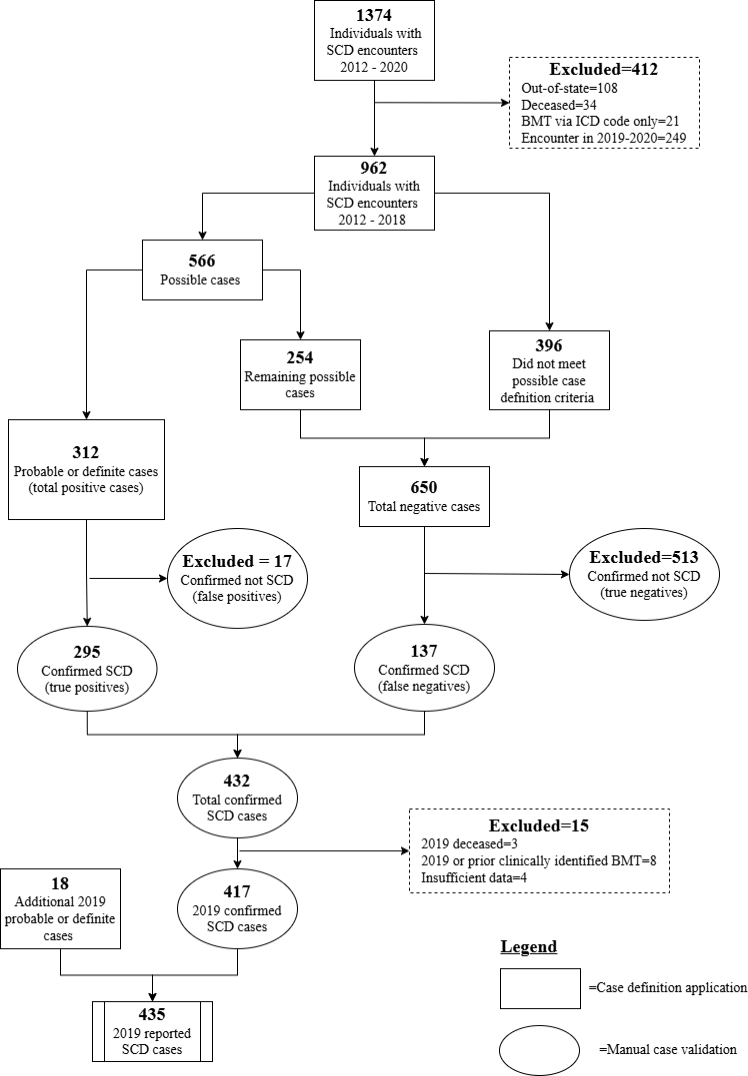
Diagram showing how the SCD counts were obtained for the year 2019 based on the application of case definitions and the manual case validation process using public health surveillance data for the Colorado Sickle Cell Data Collection Program. BMT: bone marrow transplant; *ICD*: *International Classification of Diseases*; SCD: sickle cell disease.

There was a total of 1374 individuals with a SCD *ICD-9-CM* or *ICD-10-CM* code identified during the years 2012 through 2020. After excluding encounters from individuals who were not Colorado residents, those who were deceased, or those who had encounters only in 2019‐2020, as well as those with an *ICD-9-CM* or *ICD-10-CM* code for a BMT, and applying case definitions, 566 individuals (58.8%) met the criteria for a possible, probable, or definite case in 2018 and were included for clinical validation (Figure 1). A total of 312 were probable or definite cases and therefore considered positive cases through the application of the case definitions for the year 2018. Of these, 295 were identified as TP with a PPV of 94.6% (95% CI 91.5% to 96.5%). A total of 254 possible surveillance cases were also validated and combined with 396 individuals who did not meet any level of case definition criteria for a total of 650 negative cases. Of these, 513 were identified as true negatives with a NPV of 78.9% (76.5% to 81.2%). The remaining 137 false negative cases were then classified as positive cases and added to the 295 TP cases. These 432 cases were indexed and carried over as true cases for 2019 analyses.

### 2019 Surveillance Cases

Of the 432 cases carried over as validated cases for 2019, there were 3 deaths in 2019, four with insufficient data for reporting purposes, and 8 individuals who were identified via case validation to have had a BMT in 2019 or prior not identified via ICD code, leaving 417 validated definite cases from the 2018 data set. After applying the specified case definitions to the 2019 surveillance data, an additional 18 cases met probable or definite criteria for SCD in CO, bringing the 2019 total to 435 cases (Figure 1). Most of these individuals were identified as Black or African American (n=349, 80.2%) and non-Hispanic (n=406, 93.3%). The median age within this population was 19 years, with an IQR of 8 to 30 years (Table 1).

**Table 1.** The number of individuals with sickle cell disease in Colorado in the year 2019, stratified by sex, age group, race, and ethnicity based on public health surveillance data from the Colorado Sickle Cell Data Collection Program.

Demographics	Values, n (%)
Total	435 (100)
**Sex**	
Male	215 (49.4)
Female	220 (50.6)
**Age (years)**	
<10	119 (27.4)
10‐19	100 (23)
20‐29	96 (22.1)
30‐39	78 (18)
40‐49	27 (6.2)
50+	15 (3.4)
**Race**	
Black or African American	349 (80.2)
Other	75 (17.2)
Unknown	11 (2.5)
**Ethnicity**	
Hispanic	18 (4.1)
Non-Hispanic	406 (93.3)
Unknown	11 (2.5)

### 2019 SCD Geographic Prevalence

**Figure 2.**
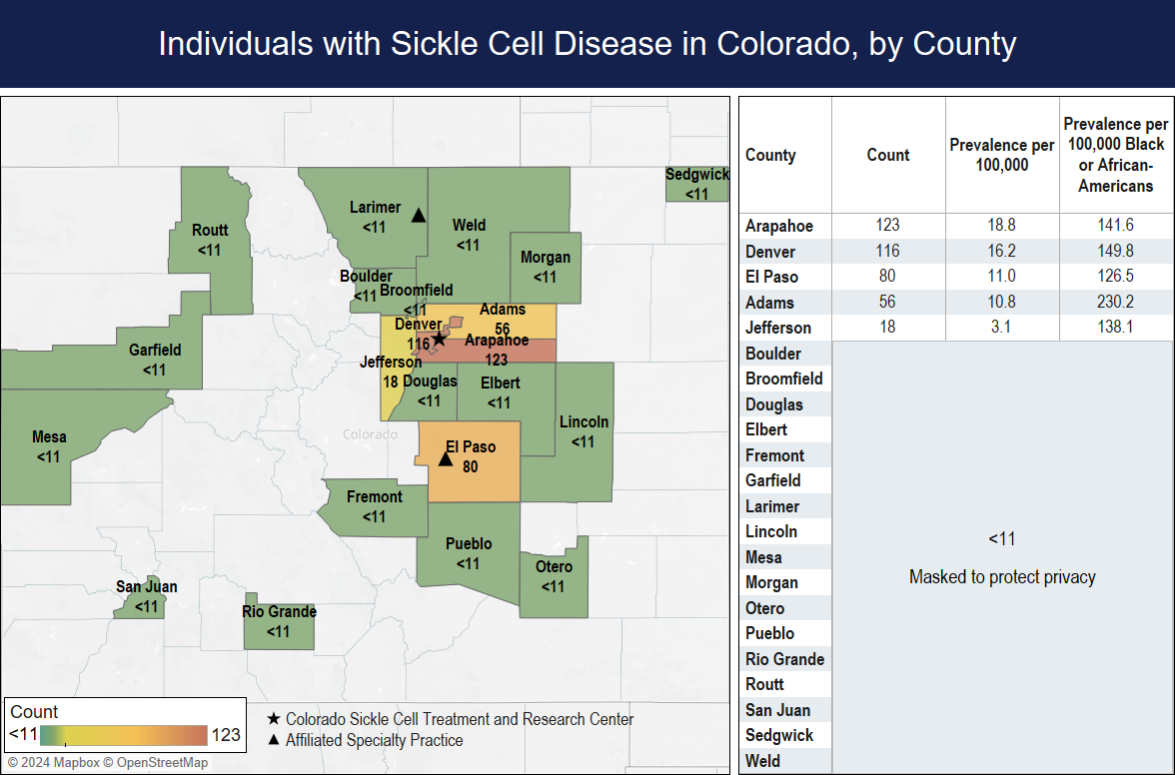
Map of sickle cell disease counts and prevalence per 100,000 individuals and per 100,000 Black or African American individuals in Colorado counties in the year 2019 based on public health surveillance data from the Colorado Sickle Cell Data Collection Program. This map was created using Mapbox and OpenStreetMap in collaboration with Tableau, a copyright of Salesforce, Inc [22].

The crude prevalence of SCD in CO in 2019 was 7.6 per 100,000 residents. Most individuals lived in urban counties (424/435, 97.5%), but geographic distribution also spread to less populated counties on the western slope and eastern rural areas of CO (Figure 2). The 3 counties with the highest prevalence rates of SCD were Arapahoe (18.8 cases per 100,000 people), Denver (16.2 cases per 100,000 people), and El Paso (11 cases per 100,000 people). The county with the highest rate of SCD by race or ethnicity was Adams with 230.2 cases per 100,000 Black or African American individuals, then Denver County with 149.8 cases per 100,000 Black or African American individuals, and finally Arapahoe with 142.1 cases per 100,000 Black or African Americans (Figure 2).

### 2015 to 2019 SCD Birth Prevalence

**Figure 3.**
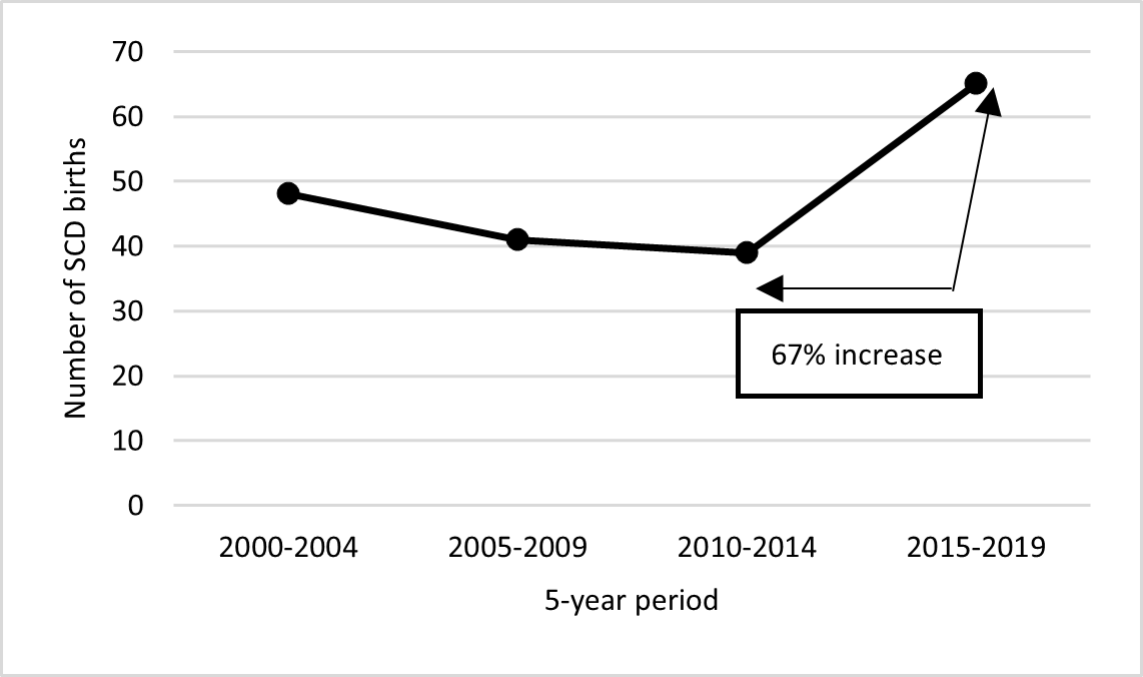
The number of SCD births during a 5-year period in Colorado increased by 67% between the years 2010 and 2019, based on public health surveillance data from the Colorado Sickle Cell Data Collection Program. SCD: sickle cell disease.

**Figure 4.**
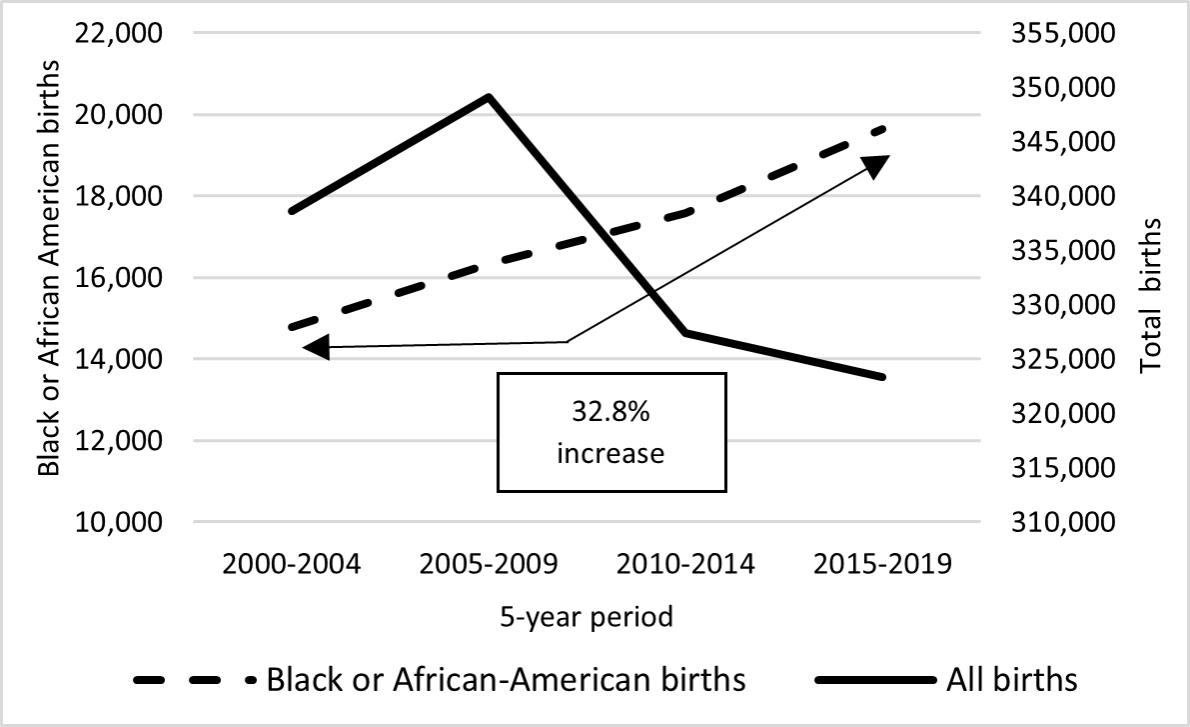
The number of Black or African American births during a 5-year period in Colorado increased by 32.8% between the years 2000 and 2019 while births across all races decreased by 4.5%, based on public health surveillance data from the Colorado Sickle Cell Data Collection Program.

There were 195 SCD births in CO between 2000 and 2019. The number of births during a 5-year period increased from 39 births between 2010 and 2014 to 65 births between 2015 and 2019 (Figure 3). The birth prevalence of SCD in CO during a 5-year period also increased from 11.9 per 100,000 live births between 2010 and 2014 to 20.1 per 100,000 live births between 2015 and 2019, equivalent to 1 in 4974 live births. When categorized by race and ethnicity, the birth prevalence among Black or African American individuals increased from 221.9 per 100,000 live births to 331 per 100,000 live births between 2015 and 2019, or 1 in 302 live births among this demographic. Congruently, the number of Black or African American live births during a 5-year period increased by 32.8% (n=3313) between the periods of 2005 and 2009 and 2015 and 2019, in contrast, the total number of all live births in CO decreased by 4.5% (n=25,782) between these same 5-year periods (Figure 4). Between 2015 and 2019, most SCD births were of the subtype hemoglobin (Hb) SS or HbSβ^0^ thalassemia (38/65, 58.5%), followed by HbSC (14/65, 21.5%), then HbSβ^+^ thalassemia (10/65, 15.4%) and other subtypes (3/65, 4.6%)

## Discussion

### Historical Estimates and Current Findings

Previously published estimates from the Agency for Health Care Policy Research estimated that approximately 640 people with SCD lived in CO as of 1993 [23]. The NNSIS used birth cohort data to estimate that between 311 and 371 individuals with SCD lived in CO between 2005 and 2007, after correcting for early mortality [6]. The CO-SCDC project found 435 individuals living with SCD in CO in 2019. Methods used for the CO-SCDC project resulted in higher counts than the NNSIS estimate by between 17% and 40%. This could be attributed to the 27.4% increase (68,367 individuals) of individuals in the Black or African American community in CO since 2010 [20,24].

The crude SCD prevalence estimate of 7.6 per 100,000 CO residents aligns with the 2021 Global Burden of Disease Study prevalence estimate of 3 to <10 per 100,000 people living in CO [25]. However, stratified by county, prevalence estimates range from 10.8 to 18.8 per 100,000 people overall and from 126.5 to 230.2 per 100,000 Black or African American residents in urban areas, underscoring the need for more precise estimates to accurately assess health care and outreach requirements for individuals with SCD in these regions.

The increase in birth prevalence supports the population growth theory as SCD births in a 5-year period increased by 67% (n=26) between 2010 and 2019, likely due to the increase of the Black or African American population in CO and the 11.7% (n=2059) increase in the birth rate among this population in a 5-year period between 2010 and 2019. Moreover, the Colorado birth prevalence of 5 to <15 per 100,000 live births, as estimated by the Global Burden of Disease Study [25], is lower than the 20.1 per 100,000 live births identified through these surveillance efforts. This suggests that the population of individuals with SCD in Colorado is growing faster than previously estimated, highlighting the need for increased resource allocation to support this population. Advancements in surveillance techniques and data methodologies used for the project likely contributed to more accurate SCD estimates within the population.

### Accessibility and Distribution of Care

The state of Colorado is home to a comprehensive SCD center, the CSCTRC, located near the epicenter of the Denver metropolitan area where approximately 70% of individuals living with SCD in Colorado reside (Figure 2). CSCTRC-affiliated providers serve patients within the major health care systems serving both children and adults in this area and provide care at an outreach clinic in the central mountain area. CSCTRC-affiliated specialty practices that see SCD patients are also located in Fort Collins and Colorado Springs, CO. This increases the coverage in the Front Range of CO to approximately 90% of individuals with SCD living in CO within a 60-mile radius of the health care systems with dedicated sickle cell specialists or affiliated specialty practices. This means around 10% of individuals with SCD in CO reside in counties on the Western Slope, Eastern Plains, or Southern CO where specialty SCD care is not readily accessible. This is important to note, as patients in these locations often require assistance to locate appropriate care facilities and to secure a provider with specialized expertise in SCD, particularly during the transition from pediatric to adult care. These factors, compounded by the scarcity of providers in rural regions willing to see sickle cell patients, are among the most frequently reported barriers to access to care among individuals with SCD [26]. The availability of surveillance data to identify areas of need informs the work of ongoing programs, such as the statewide Sickle Cell Center Transition Program, to correct these gaps.

### Surveillance and Data Methodologies

The case validation process proved crucial to the surveillance case identification process for the CO-SCDC project. Though the case definitions were very proficient at identifying true clinical cases with a PPV of 95.6%, additional validation was required as 53.9% (n=137) of possible cases were confirmed via clinical review as definite SCD cases. Had we not performed the case validation, the official surveillance estimates of SCD in 2019 would have underestimated the total count by 137 individuals (31.5%). This emphasizes the importance of manual case identification in combination with automated data processes to arrive at the most accurate results possible. While this proved a valuable and attainable goal in CO, manual case review may prove more burdensome in states with higher SCD prevalence.

Equally as important may be the ability to use claims data from administrative data sets such as Medicaid to account for the healthier population with SCD. Using EHR data exclusively may skew the data toward those who interact more frequently with the health care system, potentially underreporting healthier individuals with SCD. While CO does not currently use claims data for SCD surveillance purposes, future iterations will supplement the current data sources with all-payer claims data. This may help account for use biases and include health care encounters that may have occurred outside of the UCHealth and CHCO networks.

### Implications for Policy and Community Programs

The significance of using these data to implement change in the community cannot be overstated. The Colorado Sickle Cell Association (CSCA), the local community-based organization, plays an instrumental role in this project in disseminating and leveraging the data to tailor education and policy initiatives to address specific challenges. SCD counts reported in this paper were recently used by the CSCA to advocate before the CO General Assembly for a bill that was passed to establish an SCD outreach program to provide support to individuals living in CO with SCD [27]. Looking ahead, it is important that these data continue to expand and refine, helping the CSCA to further bridge the gap between health care providers, policy makers, and the sickle cell community.

### Limitations

This project had several limitations. Data received from Compass includes the UCHealth and CHCO network centers, hospitals, and outpatient clinics which collectively encompass the comprehensive SCD treatment center and affiliated specialty practices but do not include health care encounters from providers in the state outside of these networks, therefore, introducing bias by not accounting for out-of-network encounters and individuals who may not use either of these statewide networks. In addition, access to specific state Medicaid administrative data was not available in CO. Future analyses will incorporate all-payer claims data from the Center for Improving Value in Health Care to ensure the project is as comprehensive as possible by including data from health care encounters outside the CHCO and UCHealth networks. Additionally, there were complexities in identifying whether a SCD case still resided in CO for the reporting year and project analysts will need to find an algorithmic solution moving forward.

## Abbreviations

BMT: bone marrow transplant
CHCO: Children’s Hospital of Colorado
CO-SCDC: Colorado Sickle Cell Data Collection
CPHI: Center for Public Health Innovation
CSCA: Colorado Sickle Cell Association
CSCTRC: Colorado Sickle Cell Treatment and Research Center
EHR: electronic health record
Hb: hemoglobin
*ICD-10-CM*: *International Classification of Diseases, Tenth Revision, Clinical Modification*
*ICD-9-CM*: *International Classification of Diseases, Ninth Revision, Clinical Modification*
MDT: multidisciplinary team
NBS: newborn screening
NNSIS: National Newborn Screening Information System
NPV: negative predictive value
PPV: positive predictive value
RuSH: Registry and Surveillance System for Hemoglobinopathies
SCD: sickle cell disease
SCDC: Sickle Cell Data Collection
TP: true positive
UCHealth: University of Colorado Health
